# The promoter for intestinal cell kinase is head-to-head with F-Box 9 and contains functional sites for TCF7L2 and FOXA factors

**DOI:** 10.1186/1476-4598-9-104

**Published:** 2010-05-11

**Authors:** Thomas W Sturgill, Paul B Stoddard, Steven M Cohn, Marty W Mayo

**Affiliations:** 1 Departments of Pharmacology and Internal Medicine, University of Virginia, 1300 Jefferson Park Avenue, Charlottesville, Virginia, 22908, USA; 2 Digestive Health Center of Excellence, University of Virginia, 409 Lane Road, Charlottesville, Virginia, 22908, USA; 3 Department of Biochemistry and Molecular Genetics, University of Virginia, 1300 Jefferson Park Avenue Charlottesville, Virginia, 22908, USA

## Abstract

**Background:**

Intestinal cell kinase (ICK; GeneID 22858) is a conserved MAPK and CDK-like kinase that is widely expressed in human tissues. Data from the Cancer Genome Anatomy Project indicated ICK mRNA is increased in cancer, and that its expression correlated with expression of mRNA for an uncharacterized F-box protein, FBX9 (GeneID: 26268). *ICK *and *FBX9 *genes are arranged head-to-head on opposite strands, with start sites for transcription separated by ~3.3 kb. We hypothesized ICK and FBX9 are potentially important genes in cancer controlled by a bidirectional promoter.

**Results:**

We assessed promoter activity of the intergenic region in both orientations in cancer cell lines derived from breast (AU565, SKBR3), colon (HCT-15, KM12), and stomach (AGS) cancers, as well as in embryonic human kidney (HEK293T) cells. The intergenic segment was active in both orientations in all of these lines, and ICK promoter activity was greater than FBX9 promoter activity. Results from deletions and truncations defined a minimal promoter for ICK, and revealed that repressors and enhancers differentially regulate ICK versus FBX9 promoter activity. The ICK promoter contains consensus motifs for several FOX-family transcription factors that align when mouse and human are compared using EMBOSS. FOXA1 and FOXA2 increase luciferase activity of a minimal promoter 10-20 fold in HEK293T cells. Consensus sites for TCF7L2 (TCF4) (Gene Id: 6934) are also present in both mouse and human. The expression of β-catenin increased activity of the minimal promoter ~10 fold. ICK reference mRNAs (NM_014920.3, NM_016513) are expressed in low copy number and increased in some breast cancers, using a ten base tag 5'-TCAACCTTAT-3' specific for both ICK transcripts.

**Conclusion:**

*ICK *and *FBX9 *are divergently transcribed from a bidirectional promoter that is GC-rich and contains a CpG island. A minimal promoter for *ICK *contains functional sites for β-cateinin/TCF7L2 and FOXA. These data are consistent with functions that have been proposed for ICK in development and in proliferation or survival of some breast and colon cancers.

## Background

The ICK gene encodes an evolutionarily conserved Ser/Thr kinase in the CMGC group of the kinome, clustering in a subgroup with closely related MAK (male germ cell-associated protein kinase) and more distantly related MOK (MAPK/MAK/MRK overlapping kinase) [1]. ICK was first identified and named MRK (MAK-related protein kinase) after cloning of its cDNA from heart [2]). ICK expression was higher in the embryonic myocardium during organogenesis than in the adult tissue [2]. Decreasing expression of ICK in Colo205 cells stops proliferation and causes cell cycle arrest in G1 due to an increase in p21^Cip ^[3]. Colo205 cells greatly overexpress ICK mRNA in comparison to other lines in the NCI60, suggesting an acquired addiction to ICK for proliferation in this line. ICK mRNA is detectable in normal intestinal epithelium only in the region for lineage specification and proliferation [4].

ICK has to be phosphorylated in a TDY motif (residues 157-159) within the activation loop to be fully active. Phosphorylation of Y159 can occur by autophosphorylation, but at least phosphorylation of T157 requires transphosphorylation by another kinase [1]. ICK is a substrate for a T157-kinase related to CDK-activating kinase with gene name CCRK (cell cycle regulated kinase, [GenBank: NM_001039803]). CCRK (NM_001039803) unequivocally has T157 kinase activity because wild type but not a kinase-defective mutant phosphorylates T157 in cells [1]. Decreasing CCRK expression ~80% markedly inhibited proliferation of HCT116 and U2OS cells without a significant, specific change in G1, M, or G2/M populations but modestly increased the population with sub-G1 DNA content, suggesting increased apoptosis [5]. Other reports support a role for CCRK in molecular carcinogenesis of ovarian cancer [6]. CCRK-specific gene silencing causes ovarian cancer cells to arrest in G1 [6]. Recently, CCRK was identified as an interactor of Broadminded in Sonic hedgehog pathways [7].

## Results

### FBX9 and ICK expression are correlated genes

The NCI60 is a panel of cancer cell lines for the Cancer Genome Anatomy Project (CGAP). *FBX9 *expression correlates (R = 0.45, P = 1.5 e-04) with I*CK *expression in the NCI60 amongst genes present in microarrays from a very large collection of cDNAs. We took note because *FBX9 *is the neighboring gene to *ICK*. *FBX9 *encodes an uncharacterized F-box protein [8]. The two genes are on opposite strands, arranged head-to-head with their predicted start sites separated by only ~3.3 kb. These data suggested the intergenic segment might have bidirectional promoter activity.

We also were interested in using the intergenic segment to gain insights to ICK regulation that in turn might suggest functions. Expression of ICK mRNA is confined to the region in normal mouse epithelium where proliferation and lineage specifications occur and where β-catenin/TCF7L2 (referred to herein as TCF4) is most active. Loss of a tumor suppressor causes activation of β-catenin/TCF4 in colon cancers [9]. We hypothesized that ICK promoter activity may be increased in colon cancer cell lines (KM12, HCT-15) and in stomach cancer cells (AGS) because of this correlation. We also studied breast cancer cell lines (AU565 and SK-BR3-3) because β-catenin/TCF4 is highly active in breast cancers [10].

### The FBX9-ICK intergenic segment has bidirectional promoter activity

**Figure 1:**
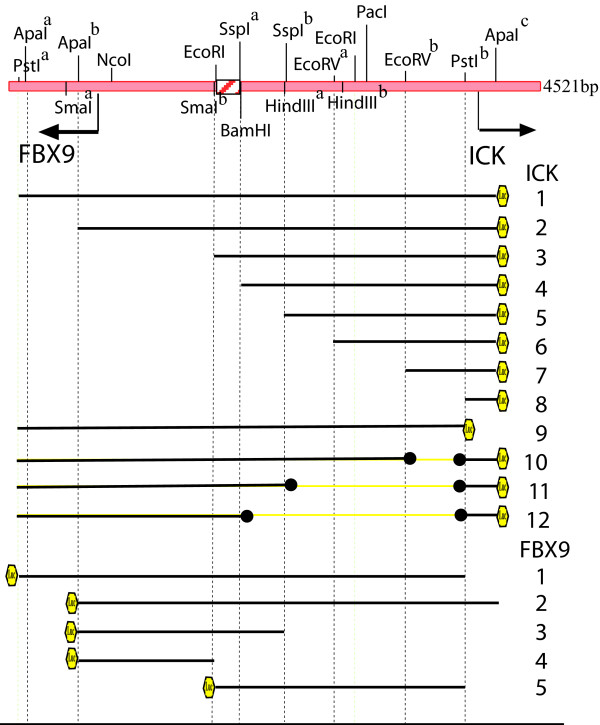
**Restriction map of genomic DNA between FBX9 and ICK and pGL3 constructs**. Indicated fragments were cloned into promoter-less pGL3 for luciferase (Luc) expression. Arrows, start of transcription for reference ICK and FBX9 mRNAs.

We obtained a clone (RP3-341E18, [GenBank: AL031178]) for a portion of the p12.3-p11.2 region of human chromosome 6 from the Sanger Institute. One XhoI restriction fragment contains the intergenic region and the start sites for transcription of both genes. This 4.5 kilobase fragment and portions thereof were placed into the promoterless pGL3-luciferase plasmid so as to generate constructs (pGL3-P_ICK_: 1-12 and pGL3-P_FBX9_: 1-5), shown schematically in (Fig. 1). We refer to constructs as ICK-1 to 12 and as FBX9-1 to 5. We used these constructs to study the promoter in five human cancer cell lines as well as in HEK293T.

**Figure 2:**
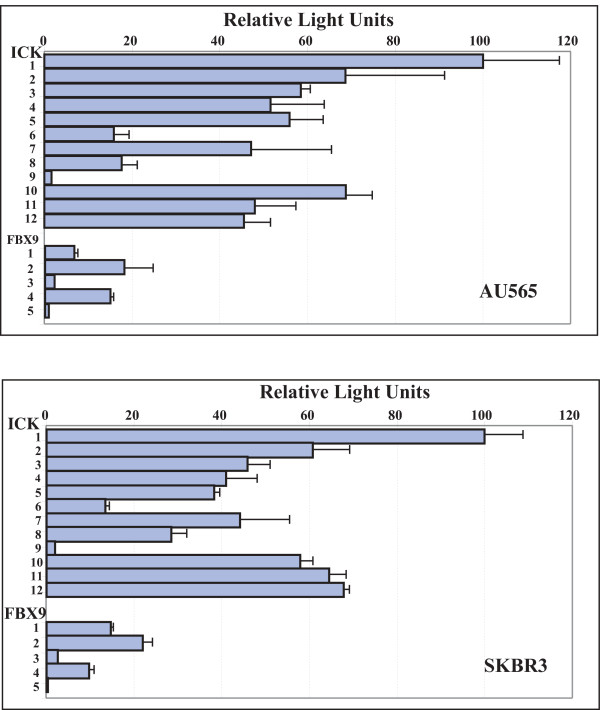
**F*BX9 *and *ICK *are divergently transcribed from a bidirectional promoter**. Equal numbers of SKBR3 and AU565 cells were seeded into 96-well plates, transfected with the indicated constructs (Figure 1), then assayed for luciferase activity in each well by the methods described. Data in figures 2 and 3 were obtained by the same procedures. Bar, ± S.D.

**Figure 3:**
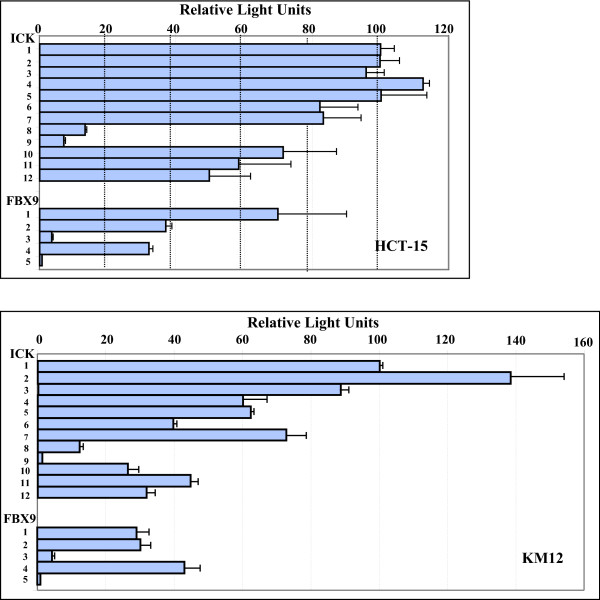
**The SspI^b ^to PstI^b ^segment contains enhancer and suppressor elements**. Equal numbers of KM12 and HCT-15 colon cancer cells were seeded into 96-well plates, transfected with the indicated constructs (Figure 1), then assayed for luciferase activity in each well by the methods described. Bar, ± S.D.

**Figure 4:**
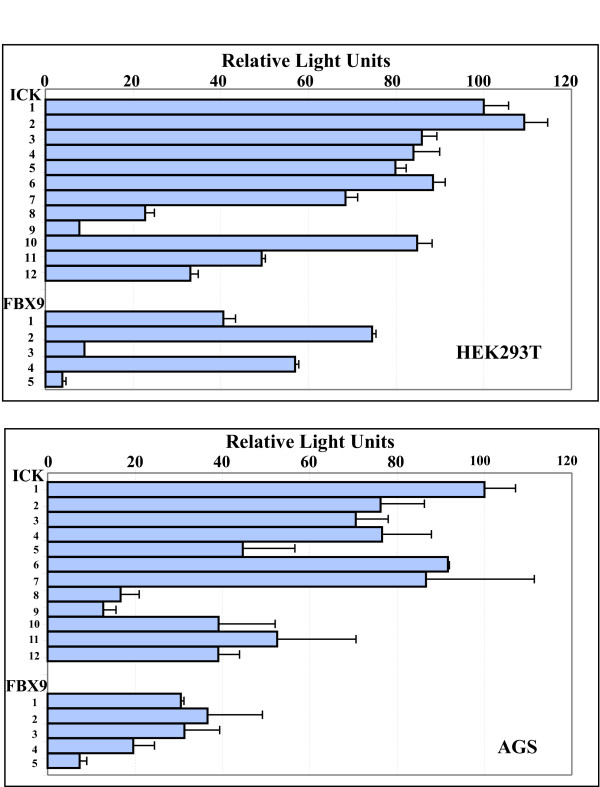
**The GC-rich EcoRV^b^-PstI^b ^segment is required for ICK reporter activity**. Equal numbers of AGS stomach cancer cells and HEK293T cells were seeded into 96-well plates, transfected with the indicated constructs (Figure 1), then assayed for luciferase activity in each well by the methods described. Bar, ± S.D.

The full intergenic segment (constructs ICK-1 and FBX9-1) was active in both orientations in all six of the lines, suggesting that ICK and FBX9 share a bidirectional promoter. Analyses in the different lines (Figs. 2, 3, 4) show elements in the common SspI^b ^to PstI^b ^fragment are important for bidirectional activity, and may account for the correlated expression of FBX9 and ICK in microarray data that motivated this study. Our analyses (Figs. 2, 3, &4) show that the intergenic segment is not a constitutive, bidirectional promoter because the FBX9 activity relative to ICK activity is variable.

### Promoter activity in HER2-overexpressing breast cancer cells

ICK promoter activity was 10-20 fold higher than FBX9 promoter activity in AU565 and SKBR3 cells, using constructs ICK-1 and FBX9-1 which contain the full intergenic segment (Fig. 2). Moreover, AU565 and SKBR3 gave similar patterns of relative activity between the different constructs derived from ICK-1 and FBX9-1. This may relate to the fact that AU565 and SKBR3 were obtained from pleural effusions from the same patient [11]. The results obtained with the truncation constructs (ICK-2 to 7) reveal enhancer elements within the SspI^b^-EcoRV^a ^segment and a suppressor element within the unique EcoRV-EcoRV fragment. The internal deletions (ICK-10 to 12) indicate another enhancer element for ICK lies in EcoRV^b^-PstI^b ^close to the ICK start site. Removal of this segment reduces ICK promoter activity 40% in both AU565 and SKBR3 cells. Extending the internal deletion from Pst1^b ^back to SspI^b ^(ICK-11), or further back to SspI^a ^(ICK-12), had modest and opposite effects. The region from SspI^b ^to PstI^b ^is particularly complex, and appears likely to have several important elements. This conclusion is borne out by data obtained from the other lines.

### Promoter activity in colon cancer cells

KM12 and HCT-15 are two colon cancer cell lines. Both are near diploid, and have relatively few structural rearrangements confined to 7 chromosomes [12]. The patterns of luciferase activity created by the constructs in these two cancer cell lines are quite different (Fig. 3). KM12 has homozygous loss for a lysine (K)-specific demethylase 6A (KDM6A); a ubiquitously transcribed X chromosome tetratricopeptide repeat protein; homozygous loss of PTEN; and heterozygous loss of p53 functions. (Some mutations in the NCI60 are available online http://www.sanger.ac.uk/). HCT-15 is null for function of APC, BRAC2, and FAM123 tumor suppressors, and has homozygous loss of p53 along with oncogenic mutations in KRAS, PI3KCα, and MSH6. The results for truncations for ICK in KM12 suggest an enhancer in SspI^b^-EcoRV^a^, and a suppressor in the unique EcoRV-EcoRV segment, and provide strong evidence for an enhancer in EcoRV^b ^- PstI^b^. The internal deletions for ICK (ICK-10 to 12) also strongly support this enhancer. Specific removal of EcoRV^b ^- PstI^b ^with ICK-10 caused a large decrease in activity, and this phenomenon was observed to different degrees in all six lines. Extending the internal deletion to SspI^b ^(ICK-11) or to SspI^a ^(ICK-12) resulted in modest changes by comparison. The largest change in activity in HCT-15 occurred with deletion of EcoRV^b^-PstI^b^.

### Promoter activity in AGS gastric cancer and HEK293T kidney cells

AGS is a human gastric cancer line that robustly expresses ICK mRNA [4]. HEK293T cells are human embryonic fibroblasts that were originally immortalized by transformation with sheared adenovirus [13], and much later made to express the large T antigen of SV40. AGS is similar to KM12 in pattern of luciferase activity between constructs, and HEK293 is similar to HCT-15 (Fig. 4). Results from AGS, like KM12 discussed above, support regulatory elements within ApaI^a^-ApaI^b^, and confirm the enhancer in SspI^b^-EcoRV^a ^and the suppressor in the unique EcoRV-EcoRV. Overall, both the truncations and the internal deletions in AGS and HEK293 strongly support importance of EcoRV^b^-PstI^b^.

### Conserved FOX binding motifs in human and mouse ICK promoters

Promoters for ICK and FBX9 are similarly configured on mouse Chr9 in a head-to-head fashion with starts for transcription on opposite strands. Because prediction of transcription factor sites is difficult at best and there are many false positive, we looked for conserved motifs present in both mouse and human that are well characterized in literature.

A striking finding was a number of consensus motifs for fork head box (FOX) proteins. Many FOX proteins bind a conserved motif with a core of TGTTTR, where R is (G, A) [14]. Also striking was the presence of a number of aligned, conserved TG motifs (TTTGTT, TTTGTTTT, TTTTGTTTGTTTT). FOX is a large family of sequence-specific factors. Its members regulate expression of many genes involved in cell growth, proliferation, differentiation and development [15,16]. The first protein in the FOX family was the *Drosophila *gene named fork head. (Prior to year 2000, certain human FOX proteins had several aliases, as winged helix protein proteins, as hepatocyte nuclear factors (HNFs), or forkhead-related clones (FREAC). For example, FOXA1 and 2 were HNF3α and β.) The winged helix domain of FOXA (HNF3) binds optimally to a consequence WWTRTTTRYWYD sequence [17], where W is (A/T), R is (A/G), Y is (C/T), and D is (A/C/T).

**Figure 5:**
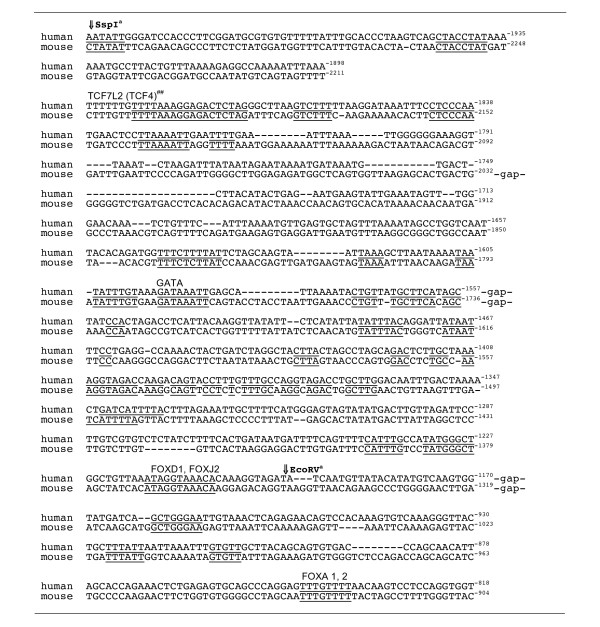
**Alignment of the human and mouse ICK promoter sequence for the SspI^a ^to EcoRV^a ^fragment**. Line numbers, distance from ICK start in human or mouse. SspI^a ^and EcoRV^a ^sites, double arrows. Underlined, motifs identical in the mouse and human. Gap, omitted bases in alignment. Putative binding sites, inserted name of the factor.

**Figure 6:**
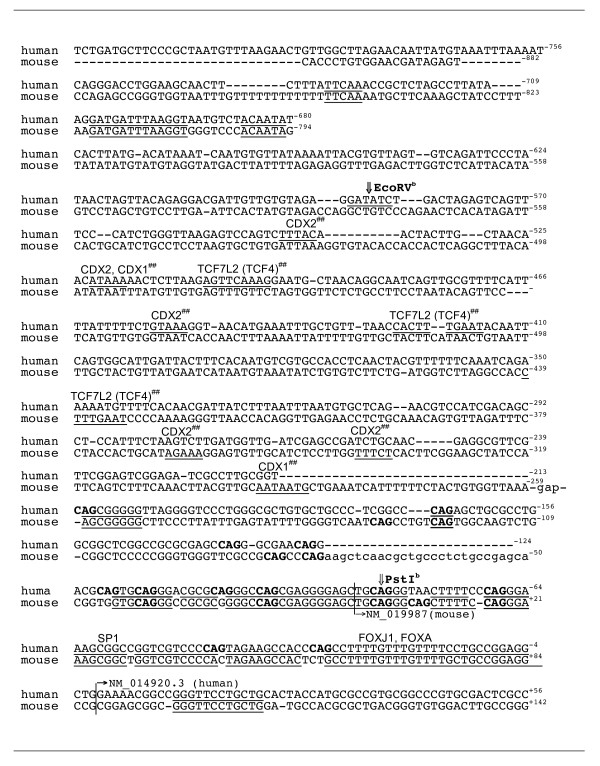
**Alignment of the human and mouse ICK promoter sequence for the EcoRV^b^-PstI^b ^fragment**. Double arrows, EcoRV^b ^and PstI^b ^sites. Single arrow, start sites of indicated reference mRNAs. Putative binding sites, inserted name of the factor.

A motif, 5'-ATAGGTAAACA-3', near -1217 nt in human ICK, is predicted to bind FOX proteins, possibly FOXD1 and FOXJ2 (Fig. 5). This motif has a conserved GTAAACA core known to bind FOXD1 (FREAC-4) and FOXF2 (FREAC-2). Bases that differentiate between family members lie near this core [14]. FOXJ2 functions in gametogenesis and early embryonic development [18]. FOXD1 functions in development of the retina [19]. A motif, 5'-GCCTTTTGTTTGTTTT-3' (near -30 nt in human), is conserved between mouse and human and contains a consensus match to FOX proteins expressed in embryonic tissues, possibly FOXJ1 or FOXJ2 (Fig. 6). This motif also matches the core for FOXA.

Beginning near PstI^b ^(Fig. 6) is a region of near identity that surrounds the transcription start sites for ICK. This region is GC-rich, and has conserved CpG sites concentrated as a CpG island. This region [NCBI gi: 1038509] was isolated in a genome-wide purification of un-methylated CpG islands [20]. CpG islands overlap the 5'end of genes, and often contain the promoter and one or more exons of genes [20]. Methylations can differentially regulate recognition by transcription factors [21]. Methylations at CpG can also change gene expression in development in set programs of activation and silencing [22], and remain as a source of epigenomic variation [23]. The putative activator of ICK, CCRK, is transcribed from a 5' start in a CpG island that is variably methylated in adult brain tissues [23].

### Minimal ICK promoter in HEK293T and HCT-15 cells

**Figure 7:**
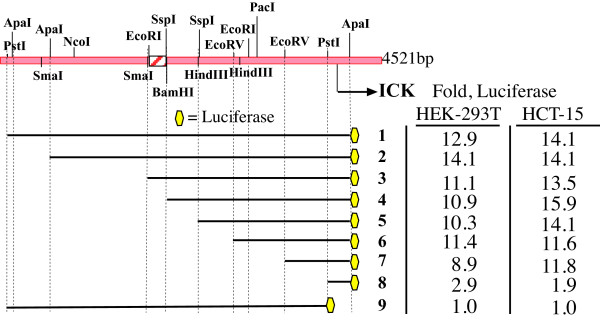
**A minimal promoter for ICK in HEK293TT and HCT-15 cells**. Data for ICK were normalized to ICK-9 construct lacking the ICK start site.

To enable initial studies of transcription factors, we chose a minimal ICK promoter for use in HEK293T cells. Activity in HEK293T (Fig. 3) and HCT-15 (Fig. 4) cells did not depend greatly on SspI^a^-SspI^b ^and SspI^b^-EcoRV^a ^fragments. To compare data from these lines, we normalized our promoter data for ICK constructs to ICK-9 (Fig. 7). Activity of the full ICK promoter (ICK-1) is increased 13-14 fold in both of these lines. The normalized results for truncations from the 5' end show that elements required for luciferase activity in HEK293T and HCT-15 cells reside in the EcoRV^a^-EcoRV^b ^(611 nt) fragment and the EcoRV^b^-Pst1 (503 nt) fragment. ICK-6 and ICK-7 also retain the majority of reporter activity for ICK in the other cell lines. The first and second EcoRV cut sites are 1195 and 587 nt, respectively, from the predicted transcription start site of human ICK. Two alternative reference mRNAs (NM_016513, NM_014920) use the same start site GGAAAAC within PstI^b^-ApaI^c^. We chose the smaller construct ICK-7 (~0.8 kb), with ~0.6 kb of 5' sequence, as the minimal promoter to study in the next experiments.

### FOXA and β-catenin activate the ICK minimal promoter in HEK293T cells

**Figure 8:**
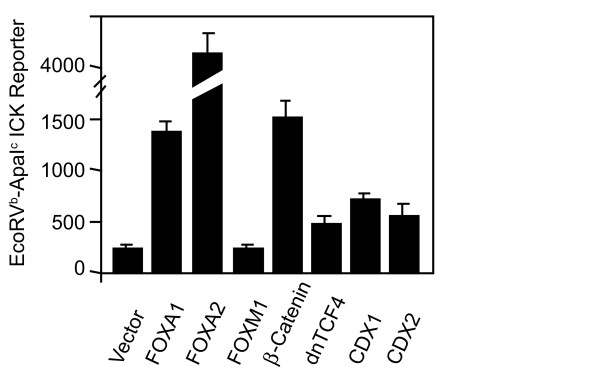
**Expression of FOXA 1, FOXA2, and β-catenin significantly increase ICK reporter activity**. HEK-293T cells in 12-well plates were transiently co-transfected with the ICK-7 luciferase reporter, the control CMV-β-galactosidase reporter, and with expression vectors encoding the various transcription factors or vector control (VC) essentially as described for NFκB reporter assays [64]. The β-galactosidase activities were used to normalize the luciferase values. Co-transfections were also performed with the promoterless ICK-9 luciferase reporter, which served as a negative transcription control. Western blot analyses were performed to ensure that cells expressed the transcription factors (see additional file 1). No suitable antibody was available for CDX1. Data represents the mean + SD of two independent experiments performed in triplicate.

We next asked if any transcription factors of importance for intestinal crypts regulate the chosen minimal promoter (ICK-7) in co-expression experiments in HEK293T. Both FOXA1 and FOXA2 caused large increases in luciferase activity (Fig. 8). FOXM1, which regulates mitotic progression, had no effect in these experiments. Western blot analyses were performed to ensure that cells expressed the transcription factors (see additional file 1). β-catenin also significantly enhanced ICK-7 activity (Fig. 8). This helps explain the presence of ICK mRNA in crypts and absence of message in the differentiated cells of the epithelium [4], but more definitive studies are necessary. Expression of a dominant-negative form of TCF4 caused a small increase in basal activity in these experiments, indicating that basal luciferase activity of the minimal reporter is not driven by β-catenin in HEK293T cells. This mutant lacks a binding site to partner with β-catenin [24].

Given the importance of TCF7L2 (TCF4) for crypt biology and colon cancer [24], we had looked for conserved TCF4 sites and failed to identify them because no TCAAG motifs were aligned by EMBOSS between human and mouse. Recently, a genome wide study for binding sites defined the majority of the *in vivo*-occupied TCF7L2-binding sites in LS174T colon cancer cells as evolutionarily conserved A-C/G-A/T-T-C-A-A-A-G motifs [25]. The motif 5'-AGTTCAAAG-3' at -539 nt (Fig. 6) is a perfect match for TCF7L2 in the human ICK promoter. The motif, 5'-CACTTTGAAT-3', at -456 nt is also a perfect match. There are also close matches for TCF7L2 motifs in the mouse ICK promoter in the same regions. These are 5'-TGCTTCAAAG-3' at -1471 nt (in a gap and not shown in Fig. 5.) and 5'-CTTTGAATC-3' (Fig. 6).

CDX1 or CDX2 plasmids increased activity insignificantly under the conditions of our experiments. CDX1 and CDX2 are distinct genes encoding related homeobox transcription factors known to have overlapping, but also distinct functions. Both CDX1 and CDX2 are expressed in crypts. Differential display identified MOK as a gene upregulated by CDX2 in stably engineered IEC-6 cells with integrated Tet/Off™ [26]. CDX1 was a much weaker activator of MOK reporter. CDX2 strongly activated a luciferase construct for the MOK promoter, and CDX2 bound to the 5' untranslated region of MOK in cells. These data prove that CDX2 regulates expression of a protein kinase related to ICK in vivo.

ICK was also characterized in sufficient detail to suggest, but not prove, that switching on CDX2 expression in also induced ICK mRNA in IEC-6 cells. This requires restudy. There are four TTTA(C/T) motifs (at -544, -522, -466, and -455) in the minimal promoter for human ICK for CDX2. Three TTTA(C/T) motifs for CDX2 are in the same region (at -275, -341, and -366 nt). A longer binding consensus was determined for chicken CDXA (CDX1) as 5'-AWTWAR-3', where W is (A/T) and D is (A/G). Motif 5'-CATAAAA-3' at -523 nt, overlaps a consensus for CDX2, and closely matches the CDX1 consensus, and is near a site for TCF7L2 (TCF4). CDX1 can interact with LEF1 on promoters [27]. An exact match for CDX1, 5'-AATAATG-3' is present at -294 nt in mouse but is not adjacent to a consensus mouse TCFL2 site. The roles of CDX1 or CDX2 if any on ICK expression in vivo are yet to be defined.

A known caveat with co-expression experiments is that activation may arise at motifs that are not motif used in the endogenous promoter. Thus, our conclusion that ICK promoter is regulated by a FOX-family protein, β-catenin, and CDX remains an hypothesis, albeit a stronger one given our data, until gel shift and site mutations *in vitro *and ChIP and knock-down experiments *in vivo *can be performed.

### ICK mRNA is increased in human cancer

Serial analysis of gene expression (SAGE) is a quantitative method to estimate copy number of a specific mRNA [28]. The SAGE method depends on identification of sequence tag(s) with high specificity for a gene. Tags from many mRNAs are isolated from polyA^+ ^mRNA, linked together, and the linked tags are sequenced. Tags appearing in the sequence are counted. The ICK 10-base tag (TCAACCTTAT) maps to the ICK gene locus and to no other locus, and is found near the 3' end of the mRNAs encoding ICK, such as isolated cDNA BC152464.1 and the reference mRNAs NM_014920.3, and NM_016513.4. ICK mRNA is >6 kb and has a 3.5 kb 3'UTR, making ICK mRNA among the top 5% in length in the human genome.

Several high quality SAGE data sets for normal breast tissue and breast cancer were available to us (Table 1). We searched each of these data sets for the ICK specific tag. The ICK transcripts are very non-abundant in breast tissue and are greatly increased in some breast cancers. No comparable studies are available with a newer 17-base SAGE tag. Microarray data for the NCI60 cancer cell lines show ICK is higher in the breast, colon, and lung cancer derived lines http://biogps.gnf.org.

**Table 1:** ICK-specific tag TCAACCTTAT in SAGE Data sets for Breast Tissuesa

Data Set^b^	Description^c^	Number, TCAACCTTAT SAGE Tags^d^	Data Set TCAACCTTAT, TAGS/Million^e^	Data Set, Total Tags in SAGE Library^f^
GSM383793	Mammary gland, DCIS-4, High Grade, Comedo	10	165	60605
GSM383790	Mammary gland, IDC, Node+, Gr3, ER+, PR+	8	119	67070
GSM383827	Metastasis to Node, from Primary Gr3, ER-, PR-	5	111	45087
GSM383796	Mammary gland, IDC-4 High Gr, Node-, ER-, erbB2-, p53+	7	109	64095
GSM383794	Mammary gland, DCIS, High Grade, Comedo	4	93	43098
GSM383795	Mammary gland, IDC-3, Low Gr, Node-, ER+, erbB2-, p53-	5	73	68891
GSM383797	Breast CA, IDC-5, Low Grade, ER+, erbB2-, p53-	4	66	60451
GSM383789	Mammary gland, IDC, Node+, Gr3 ER-, PR-,	1	25	39364
GSM383828	Metastasis to Node, from Primary Gr3, ER+, PR+	1	17	60343
GSM383824	Metastasis to Lung, Primary Gr3, ER+, PR-low, Her2-	0	N/A	49794
GSM383829	Mammary gland, Gestational Hyperplasia	1	16	61704

a SAGE data sets for libraries from tissue.

b Data set names provided by NCBI.

c Notes from clinical annotation for source and pathology.

d Absolute tag count for TCAACCTTAT in the data set.

e TAGs per million non-linker tags.

f Total tags.

### Segments important for FBX9 promoter activity

The 4.5 kb XhoI-XhoI segment (Fig. 1) contains start sites for two (*viz. *NM_016513, NM_014920) of the three reference FBX9 mRNAs. (The start site for the third (NM_012347) is ~5.5 kb distant.) Construct FBX9-2, missing the PstI^a^-ApaI^b ^fragment, was slightly more active or unchanged in comparison to FBX9-1 in four of five lines, and serves as the reference for comparison with the two 5' end deletions we were able to obtain.

HCT-15 has the highest relative FBX9 promoter activity of all six lines. Removal of the ICK half of the promoter caused a large and significant decrease (>75%) in FBX9 activity in breast (AU565, SKBR3), colon (HCT-15, KM12) as well as in HEK293T cells. Compare construct FBX9-3 to FBX9-2. Although FBX9 activities were lower than ICK activities, FBX9 activities greatly exceeded background. Since the ICK half removed contains positive cis-acting elements for ICK as well, this result is consistent with co-regulation of FBX9 and ICK. Interestingly, extending the end deletion by removal of SmaI^b^-HindIII^a ^reverses part of this loss in all the lines except AGS, suggesting repressing elements for FBX9 exist in HindIII^a^-XhoI. A repressor is one hypothesis for the differential regulation of FBX9 versus ICK in the cancer cell lines. Another is that the products (ICK and FBX9) feedback at the promoter to regulate each others expression, dependent upon the kinase activity of ICK and/or the ubiquitin ligase activity of a hypothesized SCF complex containing FBX9.

## Discussion

The full intergenic segment (constructs ICK-1 and FBX9-1, respectively) was active in both orientations in all six of the lines, suggesting that ICK and FBX9 share a bidirectional promoter. Analyses in the different lines show elements in the common SspI^b ^to PstI^b ^fragment are important for bidirectional activity, and may account for the correlated expression of FBX9 and ICK in microarray data that motivated this study. Our analyses show that the intergenic segment is not a constitutive, bidirectional promoter because the FBX9 activity relative to ICK is variable. This report extends our knowledge of ICK regulation: (i) ICK shares a bi-directional promoter with an uncharacterized F-box protein, (ii) the putative ICK 5' start is in a GC-rich region containing a CpG island that is active as a promoter, (iii) a minimal promoter can be regulated by expression of FOXA and β-catenin.

ICK is conserved and almost all metazoans and some unicellular species (including *S. pombe*) have homologs of both MAK and ICK. Human ICK/MRK and human MAK are nearly identical in the kinase domain. *Danio rerio *has one gene that encodes a protein more similar to ICK than MAK. This genome is an anomaly, as other teleost fishes have both ICK and MAK genes. ICK message is highly expressed in developing retina in zebra fish (ZDB-GENE-030131-7279). Interestingly, ICK or MAK expression is greatly increased in retinal cancer compared to normal retina (SAGE/cDNA Virtual Northern) according to data at the Cancer Genome Anatomy Project).

Our prior work established ICK as the prototype for a group of CDK and MAPK like protein kinases regulated by phosphorylation in a TDY motif [1,29]. No canonical MAP kinase cascades have yet emerged for activation of ICK, in its limited study. An alternative mechanism is transcriptional regulation followed by activation by active protein kinases. The ICK homolog in *S. cerevisiae *is regulated by transcription, and is subsequently phosphorylated in the TXY motifs dependent upon yeast CAK [29].

In an insightful commentary, Adachi and Lieber [30] noted that of twenty, functional bidirectional promoters reported in the literature at the time, several directed transcription of genes implicated in DNA repair: including BRCA1/NBR2, DNA-PKcs/MCM4, ATM/NPAT, DHFR/MSH3, and Ku86/TERP. While not unique to this class, they concluded placement of genes into bidirectional promoters is a common scenario for DNA repair genes. Clearly, this correlation does not imply anything about function of FBX9 or ICK. Nevertheless, this is of interest since ICK has interactors that may have some role in DNA repair [29].

FBX9 is predicted to encode an F-box protein [31]. F-box proteins contain a conserved domain that interacts directly with Skp1 as one of the components of a SCF (Skip, Cullin, F-box) ubiquitin ligase. The F-box protein provides a specific interaction that specifically recruits a substrate, possibly in a specific form (phosphorylated or un-phosphorylated) for degradation by linkage to ubiquitin. The substrate specificity of FBX9 is unknown.

FBX9 could produce three forms (403-447 residues) based on predicted transcripts. FBX9 has a possible homolog in *S. cerevisiae *named Hrt3p (E = 2.9e-14 over 334 residues), discovered in a single genome search of *S. cerevisiae *using SSEARCH http://fasta.bioch.virginia.edu. Reciprocally, a search of NCBI human reference proteins with Hrt3p using SSEARCH finds FBX9 as the very first hit. Hrt3p is a putative nuclear ubiquitin ligase component based on large-scale studies (see Saccharomyces Genome Database (Stanford University, Leland, CA). Hrt3p interacts with Cdc53p and Skp1p by affinity capture mass spectrometry [32], and shows dosage lethality with *cdc34*.

The intestinal epithelium has advantages for studies of differentiation, one being the segregation of the epithelium into defined zones containing stem cells, zones for proliferating transit cells, and a zone of non-proliferating differentiated enterocytes [33]. Other differentiated progeny, enteroendocrine cells, goblet cells and Paneth cells, derive from the same stem cells and assume characteristic positions in the epithelium. The epithelium is also constantly turned over during adult life. Since transcription factors regulate differentiation and are relatively easy to study, a large fund of knowledge existed for transcription factors in the gut that could suggest functions for ICK. This was a major motivation for our study. We found that FOXA1 and FOXA2, β-catenin activate an ICK reporter. These factors are known to regulate proliferation and differentiation in the intestinal epithelium [24,34,35].

Recently, mutation of ICK was linked to neonatal deaths in humans. A study of a cohort of malformed newborns in Old Order Amish families revealed R272Q mutation of ICK as the probable cause of a severe recessive, endocrine/cerebro/osteodysplasia (ECO) syndrome [36]. R272Q mutation causes loss of nuclear localization and kinase activity of ICK [1,36]. Abnormalities occurred in multiple systems, including bone, brain, and endocrine tissues [36].

If the R272Q mutation in ICK can be confirmed as causally related to the ECO syndrome, ICK is unequivocally required for normal development. The finding warrants testing a similar knock-in mutation in mouse. MAK has been knocked out in mice with no phenotype noted except for reduced fertility and reduced sperm motility [37]. Lack of a clear phenotype for a MAK knockout may be due to presence of ICK. However, the mild motility phenotype mentioned for sperm may be significant.

A single ICK/MAK homolog (LmxMPK9) in *Leishmania mexicana *regulates morphogenesis of the flagella [38]. Loss of LmxMP9 causes elongated flagella whereas overexpression of LmxMPK9 causes shortened or no flagella [38]. Genetic studies of flagella morphogenesis in *Chlamydomonas reinhardtii *identified a CCRK homolog as well as a homolog of MOK [39] as having function in flagellar morphogenesis.

These links to flagella phenotypes seem abstruse for human disease except for the fact that there is a major developmental pathway in cells that respond to Sonic hedgehog that depends on primary cilia [7]. CCRK interacts with Broadminded (Bromi) in the Sonic hedgehog pathway. We believe the cluster of genes ICK, MAK, and MOK may be regulated by CCRK and play a role in Sonic hedgehog signaling that was preceded in evolution by roles in flagellar morphogenesis in unicellular eukaryotes.

Another possible function for ICK is cell cycle regulation. The related kinase in budding yeast Ime2p (inducer of meiosis 2) controls a checkpoint that times meiotic S-phase and controls meiotic progression [40,41]. ICK can affect the cell cycle since reducing its expression in Colo205 cells causes arrest in G1 [3].

The interactors suggest leads for ICK function to the degree that the functions of the interactors are understood [29]. One interactor is multifunctional PP5, a protein phosphatase that recognizes substrates by a docking domain. The best established roles of PP5 are in control of apoptosis by inhibition of ASK1 [42]; in the cell cycle by suppressing a pathway regulating the expression of p21(waf1) [43]; in DNA repair by dephosphorylation of substrate DNA-PK [44]; and in ATR-mediated checkpoint activation via an unknown substrate [45].

The second ICK interactor we identified is the protein in literature BAT3 (NCBI designation) or Scythe or BAG6, whose functional roles are becoming clearer even if its names are not. All three names are common. ICK phosphorylates BAT3/Scythe at T1080 in vitro and in situ [29]. BAT3 functions demands more study. The name Scythe came from ability of the protein to bind reaper in *in vitro *capture experiments [46], leading to several reports supporting the idea that BAT3 functions in apoptosis [47]. BAT3, for example, can interact with an inter-membrane mitochondrial protein apoptosis-inducing factor, which seemed to fit the apoptosis-function hypothesis [47]. A Deletion of BAT3 (-, -) does cause lethality and major abnormalities in development, and not surprisingly increased apoptosis in tissues. This is also consistent, but increased apoptosis may result indirectly, not because of a proposed model that BAT3 is a direct apoptotic regulator. BAT3 (-, -) fibroblasts are not very different from wild type fibroblasts in propensity to apoptose except to a very few stimuli. BAT3 is not directly functioning in any known apoptosis cascades. A second literature supports function of BAT3 as a co-chaperone with Hsp70 and regulation of protein stability and ubiquitin-dependent degradation [48]. The kinases ICK, MAK, and MOK bind a chaperone Cdc37/p50, a nonexclusive partner of Hsp90 [49].

Finding many interactions for BAT3 suggests a scaffolding domain. We believe a unifying hypothesis for the defects in development in the BAT3 (-, -) mouse may come in the future from vigorous study of its nuclear functions. BAT3 contains a nuclear localization sequence [50]. Recent work establishes that nuclear retention of BAT3 can be dependent upon cellular transformation [51]. In the nucleus, BAT3 and SET1A form a complex with Boris to modulate H3K4 histone dimethylation marks and gene expression [52]. The latter discovery fits nicely with nuclear localization of BAT3 and transformation, abnormalities in development, and the high expression of BAT3 and MAK that occurs during spermatogenesis [53,54]. H3K and H3K4 methylation interplay to regulate gene activation [55]. Nuclear function of BAT3 is also indicated by its requirement for p53 acetylation in response to DNA damage [56]. Certain BAT3 genetic variations are strongly linked to susceptibility to lung cancer [57].

## Conclusion

ICK is transcribed from a GC-rich promoter that contains a CpG island, and shares a bidirectional promoter with FBX9. A minimal ICK promoter is activated by transcription factors (FOXA and β-catenin) that regulate proliferation and differentiation in the intestinal epithelium, motivating additional studies in vivo. Several of the candidate motifs for FOX-family proteins are conserved between mouse and human.

## Methods

### Cell lines

All of the cell lines were obtained from the American Type Culture Collection (ATCC) in Manassas, VA except the AGS cells (gift of Dr. Anil Rustgi, University of Pennsylvania). Cells were maintained in flasks (37°C) in Dulbecco's modified Eagle's medium (DMEM) supplemented with 5% fetal calf serum in an atmosphere containing 5% CO_2_. For experiments, cells were seeded into 96-well plates and allowed to attach and recover prior to transfection.

### Plasmids and cloning

BAC clone RP3-341E18 was obtained from the Sanger Gene Institute (UK). The 4.5 kbp XhoI fragment of RP3-341E18 containing the ICK and FBX9 promoter region was subcloned into the XhoI site in pBSII KS. A portion of XhoI-XhoI in pBSII KS plasmid was cloned into the promoter-less pGL3 fire fly reporter plasmid (Promega) to generate constructs shown (Fig. 1), and all of the constructs obtained were verified by sequencing or diagnostic restriction digests. Robert Costa (deceased, see tribute and commentary on his contributions to the field [58]) sent plasmids (FOXA1, FOXA2, and FOXM) [59]. Juan Iovanna (INSERM, Marseille, France) provided plasmids for human CDX1 and CDX2 [60]. Marc van de Wetering (Hubrecht Institute, Utrecht, Netherlands) gave us plasmids to express β-catenin and dominant-negative TCF4. We used MacVector software for analyses of DNA. We used Qiagen™ kits to purify DNA for transfection, and determined DNA concentration by optical density.

### Western blotting

Anti-FOXA1/HNF3α and anti-FOXA2/HNF3β were rabbit polyclonal antibodies [61]. Anti-FOXM1 was from Cell Signaling Technology. Anti-hemagglutinin antigen (HA) peptide antibody used for detection of HA-tagged β-catenin and HA-tagged dominant-negative TCF4 was obtained from Santa Cruz Biotechnology. Anti-CDX2 was a generous gift from Nathalie Rivard [62]. Anti-tubulin (Sigma Aldrich) was used as a control. No suitable antibody was available for untagged CDX1.

### Alignment of promoters and transcription factor bioinformatics

EMBOSS in setting 'needle' was used to align the human and mouse FBX9-ICK intergenic regions promoters http://www.ebi.ac.uk. We used literature and three online sites for analysis of transcription factor motifs free to academicians: Consite [63] from the Karolinska Institute, TESS [63] from the University of Pennsylvania, and Patch for TransFac™ 6 at http://www.gene-regulation.com.

### Assays

For 96-well assays, equal numbers of cells (1-2 × 10^4^) were seeded into wells and allowed to recover in 200 microliters of medium per well. Each luciferase construct (200 ng DNA/well), along with 10 ng DNA/well of control SV40-Renila luciferase plasmid (phRL-SV40 (Promega), was transfected into cells (after seeding and recovery in 200 microliters of medium per well) using *Trans*It™-LT1 reagent (Mirus Corp.). Two days after transfection, both luciferase activities were detected with Dual-Glo luciferase assay reagent (Promega), and measured by a Veritas™ micro luminometer (Turner) that has a dynamic range of greater than nine decades. The values of fire fly luciferase activity were normalized by control Renilla luciferase activity for each well. Each measurement was shown as means +/- SD of triplicate cultures and transfections. Relative Light Unit (RLU) is defined as firefly luciferase activity divided by renilla luciferase activity times ten. Data in RLU were normalized to construct ICK-1 (100%) for most comparisons; exceptions are described in the figure legends. Data are representative of multiple experiments.

## Competing interests

The authors declare that they have no competing interests.

## Authors' contributions

TWS conceived the project, obtained the Sanger clone, directed the project, performed all of the bioinformatics analyses, interpreted the data, and wrote the manuscript. PBS and MWW performed the co-expression experiments in HEK293T cells. SMC provided the motivation to embark on studies of ICK, provided expertise on roles of β-catenin in intestinal epithelium, and helped supervise the project. All authors read and approved the final manuscript.

## Supplementary Material

Additional file 1 **Western Blot**. Whole cell lysates of HEK293 cells were analyzed for protein expression from the transfected plasmids. Click here for file
