# Supplementary figures and images for: The promoter for intestinal cell kinase is head-to-head with F-Box 9 and contains functional sites for TCF7L2 and FOXA factors
Source: Mol Cancer. 2010 May 11;9:104. doi: 10.1186/1476-4598-9-104 (PMC2876993; doi:10.1186/1476-4598-9-104)

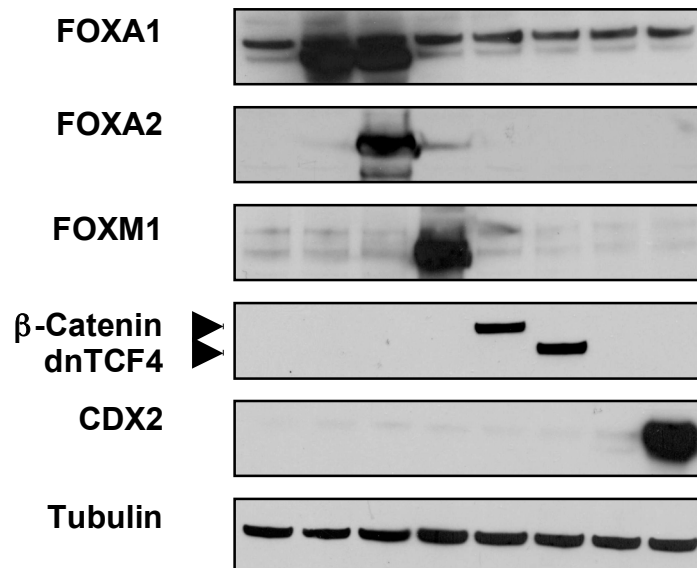

Supplement: Additional file 1 — Western Blot. Whole cell lysates of HEK293 cells were analyzed for protein expression from the transfected plasmids. [file 1476-4598-9-104-S1.PDF]
